# Testing the Water: Osteoporosis Management in Primary Care

**DOI:** 10.7759/cureus.21082

**Published:** 2022-01-10

**Authors:** Sindhuja Jothimurugan, Deepali Sanganee, Subramanian Jothimurugan, Sharmistha Williams, Myo Lynn, Arumugam Moorthy

**Affiliations:** 1 Medicine, University Hospitals of Leicester NHS Trust, Leicester, GBR; 2 Medicine, College of Life Sciences, University of Leicester, Leicester, GBR; 3 Clinical Pharmacist, Maylands Healthcare, London, GBR; 4 General Practitioner, Maylands Healthcare, London, GBR; 5 Rheumatology, Barking, Havering and Redbridge University Hospitals NHS Trust, London, GBR; 6 Rheumatology, College of Life Sciences, University of Leicester, Leicester, GBR; 7 Rheumatology, University Hospitals of Leicester NHS Trust, Leicester, GBR

**Keywords:** guidelines, rheumatology, osteoporosis, management, primary care

## Abstract

Introduction: Osteoporosis is a common bone condition in the United Kingdom (UK). The risk of osteoporosis and fragility fractures increases with age, and with the ageing population in the UK, the incidence is growing. It is imperative that General Practitioners (GPs) correctly diagnose and manage their patients with osteoporosis. To improve the awareness, a treatment pathway was developed in secondary care to guide local GPs. The aim of this study was to investigate whether patients at a GP practice with a population of 14,000 have been appropriately identified, coded as osteoporosis, treated, and have followed the recommended treatment pathway.

Methods: This retrospective study identified three patient groups through a search of the practice IT system, using the words ‘osteoporosis’, ‘fragility fracture’, ‘Quality and Outcomes Framework’, and names of all medications that are used to treat osteoporosis. Group 1 consisted of patients currently on the practice osteoporosis register. Group 2 consisted of patients with a coding of ‘osteoporosis’ or ‘fragility fracture’, but not currently on osteoporosis treatment. Group 3 consisted of patients currently on osteoporosis treatment with no coding for ‘osteoporosis’ or ‘fragility fracture’.

Results: In Group 1, 62% were found to be following the local treatment pathway in the first cycle of the study, and 70% in the second cycle. In Group 2, 45% were found to be following the local treatment pathway in the first cycle of the study, and 43% in the second cycle. In Group 3, 86% were found to be following the local treatment pathway in the first cycle of the study, and 96% in the second cycle. The completed study cycle shows an improvement of adherence of the pathway, from 75% in the first cycle to 81% in the second cycle. The first cycle of the study was presented at the GP practice meeting, which improved the awareness of the treatment pathway.

Conclusion: This study illustrates that there is a need for improvement in the diagnosis and management of osteoporosis in primary care. This can be achieved by improving awareness through continuing medical education about following the appropriate pathway to enhance the management of osteoporosis. Resources need to be allocated for prioritising osteoporosis care to prevent falls and fragility fractures, which have devastating effects on individual patients and the healthcare system.

## Introduction

Osteoporosis is characterised by low bone mass and deterioration of bone tissue, resulting in increased susceptibility to fracture [1]. It is a common bone condition in the United Kingdom (UK), with a prevalence of over 3 million, and causing over 500,00 fragility fractures each year [2,3]. Fragility fractures, the clinical consequence of osteoporosis, are fractures resulting from low-level trauma, often due to low bone density [4]. The most common fragility fracture sites are the hip, vertebrae, and wrist [5]. These fractures cause severe pain and disability, which can lead to a reduced quality of life [4]. Furthermore, hip fractures are associated with reduced life expectancy, with 10% mortality within 30 days and 33% mortality within a year [6]. The annual cost of fragility fractures to the National Health Service (NHS) is estimated to be £4.4 billion in 2010, rising to £5.5 billion in 2025 [7].

There are a number of risk factors for developing osteoporosis, including age, female gender, post-menopause, reduced body mass index, vitamin D and calcium deficiency, and long-term use of medications such as glucocorticoids and cancer chemotherapy agents [8]. The 10-year probability of an osteoporotic fracture can be calculated using an algorithm named FRAX, which assesses fracture risk using a patient’s age, body mass index, and various risk factors [9]. Osteoporosis is usually diagnosed using dual-energy x-ray absorptiometry (DEXA) scan, which measures bone mineral density. The T-score found on a DEXA scan signifies the number of standard deviations from which the bone mineral density in an individual differs from the mean bone mineral density in healthy individuals. Osteoporosis is defined as a T-score less than 2.5 standard deviations from the mean value [10]. Osteoporosis is most commonly treated using bisphosphonates, such as alendronate and risedronate, but other medications are also used, such as the human monoclonal antibody denosumab and the parathyroid hormone analogue teriparatide [1]. 

The risk of osteoporosis and fragility fractures increases with age [11], and with the ageing population in the UK, the incidence is growing. It is imperative that General Practitioners (GPs) can correctly diagnose and manage their patients with osteoporosis. Osteoporosis has been a low priority compared to other medical conditions [12]. To improve the awareness, a consultant rheumatologist at Queen’s Hospital Romford developed a treatment pathway to guide local GPs considering the high proportion of elderly residents served by the hospital [13]. 

The aim of this study was to investigate whether patients at a GP practice with a population of 14,000 have been appropriately identified, coded as osteoporosis, treated, and have followed the recommended treatment pathway. This article was previously presented as a poster at the British Journal of General Practice Research Conference 2020 on March 12, 2020.

## Materials and methods

This retrospective study identified three patient groups through a search of the practice IT system (EMIS), using the words ‘osteoporosis’, ‘fragility fracture’, ‘Quality and Outcomes Framework’, and names of all medications that are used to treat osteoporosis. Patients were excluded if they had incorrect coding. Group 1 consisted of patients currently on the practice osteoporosis register. Group 2 consisted of patients with a coding of ‘osteoporosis’ or ‘fragility fracture’, but not currently on osteoporosis treatment. Group 3 consisted of patients currently on osteoporosis treatment with no coding for ‘osteoporosis’ or ‘fragility fracture’.

The study period was from July 2017 to October 2019. Table 1 summarises the time period in both cycles of the study for patient identification, data collection, data analysis, and data comparison to the treatment pathway.

**Table 1:** Study Period

	Patient identification	Data collection	Data analysis	Data compared to the pathway
First cycle	July 2017	July-December 2017	January-May 2018	September 2018
Second cycle	July 2019	August-October 2019	October 2019	October 2019

Data was collected from electronic medical records, including age, gender, previous fragility fractures, and DEXA scans, and management. The data collection assessed whether patients were taking bone-sparing medications for osteoporosis management for the recommended time period of 3-5 years. Data was then analysed using Microsoft Excel and compared to the treatment pathway. Follow-up bone mineral density assessment was organised based on the clinical needs of patients, with the advice of secondary care.

The Barking, Havering and Redbridge University Hospitals NHS Trust (BHRUT) Pathway for initiating treatment for osteoporosis is the standard used in this study. As per the pathway, patients aged over 50 who have had a fragility fracture should be considered for a DEXA scan, their vitamin D and risk factors should be optimised, and blood tests for thyroid function tests, parathyroid hormone, coeliac screen, prostate-specific antigen, and myeloma screen should be carried out. For patients whose DEXA scan shows a T score of less than -2.5, or for patients not able to tolerate a DEXA scan, oral alendronate 70 mg per week should be started. Patients who are intolerant of alendronate can be started on oral risedronate 35 mg per week or subcutaneous denosumab. Patients should have a repeat DEXA scan after 3-5 years. If this scan shows normal bone mineral density, treatment should be stopped, with continuation of calcium and vitamin D and lifestyle modifications. If the scan shows deteriorating bone mineral density, patients should be referred to secondary care. Patients who have clinical risk factors for osteoporosis should have a fracture risk assessment to calculate the 10-year probability of major osteoporotic fracture. Risk factors should then be corrected and underlying conditions treated, with reassessment then carried out. The interval for a repeat DEXA scan should be decided on a case-by-case basis. Patients should be referred to secondary care if there is any doubt about management.

## Results

For the first cycle of the study, 173 patients were identified. 22 were excluded due to incorrect coding, and the remaining patient number was n = 151. For the second cycle of the study, 103 patients were identified. Two were excluded due to incorrect coding, and the remaining patient number was n= 101. 

Group 1

In Group 1, which consisted of patients currently on the practice osteoporosis register, there were 77 patients included in both cycles of the study. There were 34 patients included in Group 1 of the first cycle of the study, of whom 34/34 (100%) were aged over 50. The age cut off of 50 years was included in the local treatment pathway. In terms of gender, 4/34 (12%) of the patients were male, with 30/34 (88%) being female. 34/34 (100%) of the patients in this group had sustained a fragility fracture, and 20/34 (59%) had had a DEXA scan; 31/34 (91%) of the patients were appropriately treated, and it was found that 21/34 (62%) of patients had followed the local treatment pathway. There were 43 patients included in Group 1 in the second cycle of the study, of whom 43/43 (100%) were aged over 50. In terms of gender, 7/43 (16%) of the patients were male, with 36/43 (84%) being female; 41/43 (95%) of the patients in this group had sustained a fragility fracture, and 26/43 (60%) had had a DEXA scan; 39/43 (91%) of the patients were appropriately treated, and it was found that 30/43 (70%) of patients had followed the local treatment pathway. Table 2 summarises these results.

**Table 2:** Group 1 Results

	Total number	Age >50	Gender	Sustained fragility fracture	DEXA scan	Appropriately treated	Local pathway followed
First cycle	34 (100%)	100%	M 12%, F 88%	100%	59%	91%	62%
Second cycle	43 (100%)	100%	M 16%, F 84%	95%	60%	91%	70%

Although 91% of the patients were appropriately treated in both cycles of the study, the percentage of patients who had followed the local pathway was lower than this, with 62% in the first cycle and 70% in the second cycle. This is due to patients having been on the appropriate treatment for longer than advised by the pathway. However, in the second cycle the percentage of patients following the pathway did increase by 8%, highlighting that an improvement had been made.

Group 2

In Group 2, which consisted of patients with a coding of ‘osteoporosis’ or ‘fragility fracture’ but not currently on osteoporosis treatment, there were 29 patients included in both cycles of the study. There were 22 patients included in Group 2 of the first cycle of the study, of whom 22/22 (100%) were aged over 50. In terms of gender, 5/22 (23%) of the patients were male, with 17/22 (77%) being female. 21/22 (95%) of the patients in this group had sustained a fragility fracture, and 18/22 (82%) had had a DEXA scan; 15/22 (68%) of the patients were appropriately treated, and it was found that 10/22 (45%) of patients had followed the local treatment pathway. There were seven patients included in Group 2 of the second cycle of the study, of whom 7/7 (100%) were aged over 50. In terms of gender, 1/7 (14%) of the patients were male, with 6/7 (86%) being female. 6/7 (86%) of the patients in this group had sustained a fragility fracture, and 6/7 (86%) had had a DEXA scan; 2/7 (29%) of the patients were appropriately treated, and it was found that 3/7 (43%) of patients had followed the local treatment pathway. Table 3 summarises these results.

**Table 3:** Group 2 Results

	Total number	Age >50	Gender	Sustained fragility fracture	DEXA scan	Appropriately treated	Local pathway followed
First cycle	22 (100%)	100%	M 23%, F 77%	95%	82%	68%	45%
Second cycle	7 (100%)	100%	M 14%, F 86%	86%	86%	29%	43%

A lower proportion of patients were treated appropriately and followed the local pathway in Group 2 than in Group 1, as the majority of patients in Group 2 were non-compliant with treatment. Surprisingly, the percentage of patients treated appropriately decreased significantly from 68% in the first cycle to 29% in the second cycle. This is likely due to the patients in the first cycle being moved to the other groups after being started on treatment, and the majority of patient’s remaining in Group 2 for the second cycle having opted not to start osteoporosis treatment.

Group 3

Historically, due to the lack of awareness of coding in primary care staff and the transition from handwritten notes to electronic records, many patients were on treatment for osteoporosis without appropriate coding. In order to gain a full representation of patients with osteoporosis, Group 3 was created, which consisted of patients currently on osteoporosis treatment with no coding for ‘osteoporosis’ or ‘fragility fracture’, and there were 146 patients included in both cycles of the study. There were 95 patients included in Group 3 of the first cycle of the study, of whom 95/95 (100%) were aged over 50. In terms of gender, 46/95 (48%) of the patients were male, with 49/95 (52%) being female. 46/95 (48%) of the patients in this group had sustained a fragility fracture, and 90/95 (95%) had had a DEXA scan. 93/95 (98%) of the patients were appropriately treated, and it was found that 82/95 (86%) of patients had followed the local treatment pathway. There were 51 patients included in Group 3 of the second cycle of the study, of whom 50/51 (98%) were aged over 50. In terms of gender, 13/51 (25%) of the patients were male, with 38/51 (75%) being female; 23/51 (45%) of the patients in this group had sustained a fragility fracture, and 26/51 (51%) had had a DEXA scan; 51/51 (100%) of the patients were appropriately treated, and it was found that 49/51 (96%) of patients had followed the local treatment pathway. Table 4 summarises these results.

**Table 4:** Group 3 Results

	Total number	Age >50	Gender	Sustained fragility fracture	DEXA scan	Appropriately treated	Local pathway followed
First cycle	95 (100%)	100%	M 48%, F 52%	48%	95%	98%	86%
Second cycle	51 (100%)	98%	M 25%, F 75%	45%	51%	100%	96%

Compared to other groups, in Group 3, the proportion of male patients is higher, along with a lower proportion of patients having sustained fragility fractures. This is because many patients in this group were treated for osteoporosis as they had clinical risk factors for osteoporosis, such as being on long term steroid treatment or chemotherapeutic treatments, as opposed to being diagnosed with osteoporosis after having had a fragility fracture. Of all the groups, Group 3 had the highest proportion of patients treated appropriately and having followed the local pathway, along with an improvement between the first and second cycle of the study.

First cycle vs second cycle

Overall, there were 252 patients included in both cycles of the study. There were 151 patients included in the first cycle of the study, of whom 151/151 (100%) were aged over 50. In terms of gender, 55/151 (36%) of the patients were male, with 96/151 (64%) being female; 101/151 (67%) of the patients had sustained a fragility fracture, and 128/151 (85%) had had a DEXA scan; 139/151 (92%) of the patients were appropriately treated, and it was found that 113/151 (75%) of patients had followed the local treatment pathway. There were 101 patients included in the second cycle of the study, of whom 100/101 (99%) were aged over 50. In terms of gender, 21/101 (21%) of the patients were male, with 80/101 (79%) being female; 70/101 (69%) of the patients had sustained a fragility fracture, and 58/101 (57%) had had a DEXA scan. 92/101 (91%) of the patients were appropriately treated, and it was found that 82/101 (81%) of patients had followed the local treatment pathway. Table 5 summarises these results.

**Table 5:** Overall Results

	Total number	Age >50	Gender	Sustained fragility fracture	DEXA scan	Appropriately treated	Local pathway followed
First cycle	151 (100%)	100%	M 36%, F 64%	67%	85%	92%	75%
Second cycle	101 (100%)	99%	M 21%, F 79%	69%	57%	91%	81%

The completed study cycle shows an improvement of adherence of the pathway, from 75% in 2018 to 81% in 2019. The first cycle of the study was presented at the GP practice meeting, which improved the awareness of the treatment pathway.

## Discussion

Primary care adherence to the management of osteoporosis has been assessed, with previously published studies having identified that osteoporosis management is often not focused on in primary care. A study in Sweden [12] found that only 14% of patients were treated for osteoporosis after a fragility fracture. The study explored primary care physicians’ views and found that they regarded osteoporosis to not be a priority compared to other medical conditions, and that there was low awareness about the condition as well as low confidence level on how it should be managed. A study across eight countries in Europe [14] found that in primary care, 25.4% of women aged over 70 years with a high risk of fragility fracture were being treated appropriately for osteoporosis, which was linked to low proportion of osteoporosis diagnoses. A study in the United Kingdom [15] found that only 7.5% of patients at risk of fragility factures were diagnosed with osteoporosis, and only 42% of patients within this cohort were treated appropriately. These studies illustrate that there is an educational need for primary care clinicians to diagnose and treat osteoporosis. However, there should be enough resources allocated to improving osteoporosis care, such as the Royal Osteoporosis Society. 

There are a number of recommendations on improving diagnosis and management of osteoporosis in primary care. One recommendation is for primary care physicians to routinely evaluate osteoporosis risk in women aged over 50 years, by reviewing risk factors and using online fracture risk assessment tools such as FRAX [16]. A study reviewing the effects of patients self-referring to be reviewed for osteoporosis risk found that self-referral led to increased osteoporosis diagnosis [17]. Another study suggested the use of a bone health team, which found increased rates of DEXA scans, osteoporosis diagnoses, and osteoporosis treatment in patients who were involved with a bone health team [18]. The use of osteoporosis guidelines, improving awareness though continuing educational programmes for GPs, allocation of resources for screening and management of osteoporosis such as specialist osteoporosis nurses for primary care, community osteoporosis clinics, and fracture liaison services in Emergency Departments may go a long way in managing this preventable and treatable condition.

The completed study identifies that there was a gap in managing osteoporosis in primary care prior to the introduction of the treatment pathway created by the local secondary care team. The improvement in the proportion of patients following the local osteoporosis pathway, from 75% in 2018 to 81% in 2019, emphasises the importance of having a guideline for GPs to follow in order to optimise treatment and prevent future fragility fractures. The first cycle of the study was presented at the GP practice meeting, which improved the adherence of management of osteoporosis.

There are a few limitations to consider in this study. This is a single GP practice study with a small sample size, whereas a multicentre study may show a more accurate representation. This study was also limited by the lack of documentation in primary care notes, including why some patients did not have baseline DEXA scans to determine severity of osteoporosis or the site of fractures. Furthermore, despite efforts to include all patients with osteoporosis in the study, there is a chance that there may have been some patients excluded, who had sustained fragility fractures but were not coded appropriately or taking treatment for osteoporosis.

This study illustrates that there is a need for improvement in diagnosis and management of osteoporosis in primary care. This can be achieved by improving awareness through continuing medical education about following the appropriate pathway to enhance the management of osteoporosis. Resources need to be allocated for prioritising osteoporosis care to prevent falls and fragility fractures, which have devastating effects on individual patients and the healthcare system.

## Conclusions

Osteoporosis is a silent killer and poses a major public health problem, with fragility fractures causing morbidity and mortality, as well as a high cost to the NHS. It is important that patients with osteoporosis are treated appropriately in order to minimise this, and the study cycle highlights an improvement in appropriate treatment when the local treatment pathway has been followed. Overall, the findings support that GP surgery lists can be utilised to identify and manage patients with osteoporosis and ensure that the pathway is followed. This will help GPs to diagnose and treat patients proactively and efficiently.
